# Impacts of Microprocessor-Controlled Versus Non-microprocessor-Controlled Prosthetic Knee Joints Among Transfemoral Amputees on Functional Outcomes: A Comparative Study

**DOI:** 10.7759/cureus.24331

**Published:** 2022-04-21

**Authors:** Abdallah M Alzeer, Naresh Bhaskar Raj, Enas M Shahine, Wan-Arfah Nadiah

**Affiliations:** 1 School of Rehabilitation Science, Universiti Sultan Zainal Abidin, Kuala Terengganu, MYS; 2 Prosthetics and Orthotics, Sultan Bin Abdul Aziz Humanitarian City, Riyadh, SAU; 3 Medical Affairs, Sultan Bin Abdul Aziz Humanitarian City, Riyadh, SAU; 4 Faculty of Health Sciences, Universiti Sultan Zainal Abidin, Kuala Terengganu, MYS

**Keywords:** transfemoral amputation, amputee quality of life, microprocessor prosthetic, prosthetic knee, prosthetic rehabilitation

## Abstract

Introduction: Selecting a prosthetic knee mechanism is an important part of transfemoral (TF) amputee rehabilitation. Prosthetic knee joint selection depends on the users' gait and their energy consumption. This study compares the feedback of transfemoral prosthesis users based on the prosthetic knee design self-reporting responses using the Prosthetic Evaluation Questionnaire (PEQ) outcome measure.

Objective: This study aims to assess the impact of using a microprocessor-controlled prosthetic knee (MCPK) compared with a non-microprocessor-controlled prosthetic knee (NMCPK); feedback on the amputee usage can improve the clinical decision for proper prosthetic knee joint selection.

Methods: This is a cross-sectional study with a total of 76 adult unilateral transfemoral amputees classified into two groups. The participants in the first group (38) used the MCPK (Genium, Otto Bock, Minneapolis, MN, USA), and the participants in the second group (38) used the NMCPK (hydraulic and total knee joints). Enrolment was based on a sequence of appointments where all participants answered the PEQ, with different subscale questions including utility (UT), sounds (SO), appearance (AP), residual limb health (RL), frustration (FR), perceived response (PR), social burden (SB), ambulation (AM), and quality of life (QoL). PEQ was filled out during the follow-up appointments at the prosthetic clinic through a visual analog scale (VAS). All data entered into a database were analyzed.

Result: The MCPK participants have significantly improved utility, appearance, ambulation, and total PEQ score, the same results as the male participants. Middle-adulthood (25-40 years) MCPK participants have a significant p-value in the score of utility, frustration, ambulation, and total PEQ score compared to early-adulthood (18-24 years) and late-adulthood (41-60 years) participants. Also, there was a significant improvement in the p-value in ambulation scores in participants using MCPK with amputations caused by diseases compared to amputations caused by trauma and congenital cause.

Conclusion: Transfemoral amputee prosthesis utility, natural gait, and ambulation improved when using MCPK compared to when using NMCPK during prosthetic rehabilitation.

## Introduction

Amputees are people who have had their limb amputated as a result of various life activities or treatment procedures. An acquired amputation happens at any age due to different reasons. Others are born without limbs or have a malformation of their limbs, defined as congenital limb loss. Amputees' rehabilitation is required as part of the plan of care for both acquired and congenital amputations. Prosthetic intervention is required for amputation recovery to adapt to new difficulties and regain lost function.

The level of an amputation usually decides which joint should be replaced on the prosthesis. Transfemoral (TF) amputees require a prosthetic knee and prosthetic foot to restore functional performance and cosmetic perspectives. The design of a prosthetic knee is critical for patient mobility, stability, balance, and control, cosmetic restoration, and individual tasks such as climbing stairs. A prosthetist usually chooses a prosthetic knee joint based on the amputee's medical condition, rehabilitation goals, and financial capability [1]. There are more than 220 prosthetic knee joint designs available worldwide, with different shapes, types, prices, functionality, mobility levels, and weight categories. Control units for prosthetic knee joints are either externally powered, mechanical, hydraulic, or pneumatic [2].

Prosthetic knee joints are mainly classified according to their control in both stance and swing phases. Mechanical joints are usually controlled by constant friction on the surface of articulation, weight activation (loading and unloading), and ground reaction force related to the pivot of the joint located in the polycentric knee joint (instantaneous center of rotation), and manual lock knee. Hydraulic and pneumatic joints control flexion/extension by managing the amount of fluid (liquid or air) passing in and out of hydraulic or pneumatic units. Some hydraulic/pneumatic systems are weight-activated, polycentric, and monocentric knee joints. Mechanical joints and hydraulic and pneumatic joints are classified as non-microprocessor-controlled prostheses (NMCPK) [3].

Microprocessor-controlled prosthetic knee (MCPK) is defined as "a system that includes a computer to control a mechanism that senses user-specific needs and adapts to implement variable and complex functions" [4]. MCPK designs are not powered knees and do not have a motor to propel them forward. MCPK only controls and manages gait phases during the gait cycle. Some MCPK only controls stance phases, such as C-Leg Compact (Otto Bock, Minneapolis, MN, USA) (with program adjustment). Other MCPK types control only the swing phase, such as SmartIP (Endolite, Miamisburg, OH, USA). C-Leg and Genium (Otto Bock) and Rheo Knee (Ossur, Reykjavík, Iceland) control the stance and swing phases. MCPK Genium technology was launched in 2011. The extant literature focuses on the MCPK C-Leg, with fewer studies reporting on the effects of Genium on users [5]. The inconsistency of the results in the literature and the need for defining the differences between MCPK and NMCPK [6,7] support the need for the study.

This study aims to compare the effects of employing MCPK (Genium) versus NMCPK for hydraulic and pneumatic designs (3R80, 3R95, 3R106, 3R60, and total knee joint) while excluding mechanical designs. Current clinical practice recommendations have not been established in the field [8,9], and with considerable expertise in the prosthetic provision, the projected outcome of this investigation will impact the criteria for prosthetic knee joint selection [10]. Few studies have compared MCPK with NMCPK, and the predicted value of this comparison is not yet clarified [5]. This comparison will help us highlight the importance of use, make prescribing criteria for NMCPK and MCPK easier, and assist to match patient expectations in the assessment phase.

## Materials and methods

This is a hospital-based comparative study based on the feedback of patients who had previously been fitted with transfemoral prostheses to meet the objectives and report the impact of various prosthetic knee joints. The study was based on the quality of life outcome (QoL) that was self-reported using PEQ, consisting of scores for ambulation (AM), appearance (AP), frustration (FR), perceived response (PR), residual limb health (RL), social burden (SB), sounds (SO), utility (UT), and quality of life (QoL). The inclusion criteria were met by 76 transfemoral amputees who followed the follow-up appointments and agreed to participate in the study by signing the informed consent form. The first 38 participants used MCPK joints, while the remaining 38 used NMCPK of various varieties, including total 2000/2100, 3R60, 3R106, 3R80, and 3R95. All subjects received a post-prosthetic rehabilitation regimen that included a variety of training methods. Socket fitness was assured to meet the inclusion criteria, and no one had adjusted their prosthesis alignment in the previous eight weeks. Enrolments were based on clinical appointments rather than following any sampling method. Based on the published study [11], a sample size of 76 transfemoral participants, 38 participants per group, was calculated to ensure a type 1 error rate of 0.05 and power of analysis of 0.8. Other criteria for inclusion were age (18-60) years old, medically stable, outdoor ambulator with mobility levels K3 and K4 [12], and intact cognition. Participants with other amputations or disabilities, those weighing less than 50 kg and more than 150 kg, and pregnant women were excluded.

Data were collected using the case report form (CRF) approved by the institutional research board of Sultan Bin Abdul Aziz Humanitarian City (SBAHC), Riyadh, Kingdom of Saudi Arabia, and Universiti Sultan Zainal Abidin (UniSZA) Human Research Ethics Committee (UHREC), Kuala Terengganu, Malaysia. All participants received prostheses from SBAHC. After the consent form was signed, the validated form of PEQ [13] was introduced for each participant and answered individually. The main outcome measure was assessing the patients' satisfaction using the PEQ. Permission to use the PEQ [12] and its validated Arabic version [14] was obtained. Visual analog scale (VAS) questions have been selected as PEQ design with 41 questions. The average for each category was counted and considered as the score, based on PEQ analysis recommendation.

For all analyses, SPSS version 23.0 (IBM Corporation, Armonk, NY, USA) was used. The collected data were checked for normal distribution and analyzed using the t-test for paired samples to detect differences in the results of the two groups (p = 0.05) and Pearson correlation to detect similarities. Based on the PEQ analysis advice, the average for each category was counted, and the score was calculated.

## Results

In this study, 76 transfemoral prosthesis users were registered, with 38 using MCPK Genium and 38 using NMCPK. The participants' age ranged from 18 to 55 years old, with an average age of 33.8. All participants were divided into three age groups: 14 (18.4%) participants in the early adulthood (18-24 years), 44 (57.9%) participants in the middle adulthood (25-40 years), and 18 participants (23.7%) in the late adulthood (41-60 years). Out of the total participants, 66 (86.84%) were male, and 10 (13.16%) were female. Sixty-three (82.89%) participants were Saudi nationals, while 13 (17.10%) were non-Saudis. When considering the cause of amputation, trauma was among 49 (64.5%) participants, followed by those having a disease as the cause of amputation in 21 (27.6%) participants, and then those with the congenital cause of amputation in six (7.9%) participants (Table 1).

**Figure 1:**
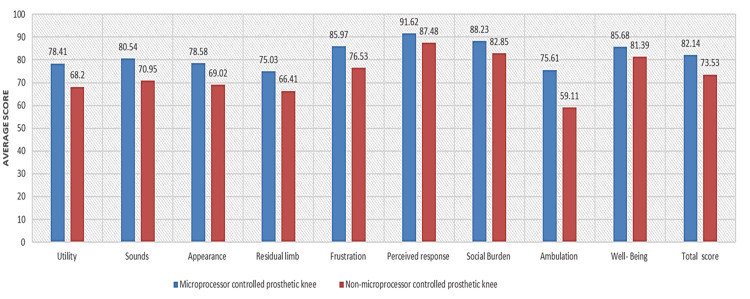
PEQ average comparison PEQ: Prosthetic Evaluation Questionnaire

Figure 1 shows that MCPK users have higher scores in all PEQ items and total. In Table 2, MCPK users improve significantly on the utility scale (p = 0.025), appearance scale (p = 0.039), ambulation scale (p = 0.04), and the total average of the nine measures (p = 0.014). In Table 3, a significant improvement was found among MCPK users in terms of utility scale (p = 0.02), followed by frustration (p = 0.04), ambulation (p = 0.001), and total average score (p = 0.012). However, no significance was found in the early- and late-adulthood groups. Table 4 shows that male participants improved significantly in terms of utility (p = 0.04), appearance (p = 0.047), ambulation (p = 0.01), and total average (p = 0.015) when compared to NMCPK participants. On the other hand, females had no significant difference between MCPK and NMCPK. Table 5 shows a significant improvement in ambulation scores among MCPK users with amputation caused by diseases (p = 0.002) compared to NMCPK from the same cause of amputation. No significance was found in participants whose amputation was caused by trauma and congenital limb loss.

**Table 1:** Demographic characteristics and amputation etiology for all studied participants MCPK: microprocessor-controlled prosthetic knee, NMCPK: non-microprocessor-controlled prosthetic knee

Categories	Subcategories	Total (N (%))	MCPK (n = 38) (N (%))	NMCPK (n = 38) (N (%))	p-value
Age	Early adulthood	14 (18.4)	4 (10.5)	10 (26.3)	0.078
	Middle adulthood	44 (57.9)	21 (55.3)	23 (60.5)	0.648
	Late adulthood	18 (23.7)	13 (34.2)	5 (13.2)	0.030
Gender	Male	66 (86.8)	34 (89.5)	32 (84.2)	0.497
	Female	10 (13.2)	4 (10.5)	6 (15.8)	0.499
Nationality	Saudi	63 (82.90	35 (92.1)	28 (73.7)	0.034
	Non-Saudi	13 (17.1)	3 (7.9)	10 (26.3)	0.034
Amputation etiology	Traumatic	49 (64.5)	27 (71.1)	22 (57.9)	0.232
	Disease	21 (27.6)	10 (26.3)	11 (28.9)	0.801
	Congenital	6 (7.9)	1 (2.6)	5 (13.2)	0.089

**Table 2:** PEQ results based on prosthetic knee joint categories PEQ: Prosthetic Evaluation Questionnaire, MCPK: microprocessor-controlled prosthetic knee, NMCPK: non-microprocessor-controlled prosthetic knee

PEQ	MCPK (mean ± SD)	NMCPK (mean ± SD)	p-value
Utility	78.41 ± 16.22	68.20 ± 22.18	0.025
Sounds	80.54 ± 26.2	70.95 ± 30.88	0.149
Appearance	78.58 ± 19.54	69.02 ± 20.18	0.039
Residual limb	75.03 ± 21	66.41 ± 22.94	0.092
Frustration	85.97 ± 24.67	76.53 ± 27.15	0.119
Perceived response	91.62 ± 13.04	87.48 ± 16.37	0.226
Social burden	88.23 ± 17.81	82.85 ± 21.51	0.241
Ambulation	75.61 ± 22.09	59.11 ± 24.06	0.003
Well-being	85.68 ± 17.95	81.39 ± 23.95	0.38
Total average score	82.14 ± 14.92	73.53 ± 14.8	0.014

**Table 3:** PEQ for age groups based on their prosthetic knee joint type PEQ: Prosthetic Evaluation Questionnaire, MCPK: microprocessor-controlled prosthetic knee, NMCPK: non-microprocessor-controlled prosthetic knee

PEQ	Prosthetic knee	Early adulthood		Middle adulthood		Late adulthood	
		Mean ± SD	p-value	Mean ± SD	p-value	Mean ± SD	p-value
Utility average	MCPK	80.59 ± 6.86	0.346	79.64 ± 17.91	0.020	75.76 ± 15.92	0.832
	NMCPK	74.99 ± 14.38		64.07 ± 24.08		73.63 ± 25.46	
Sounds average	MCPK	61.25 ± 43.28	0.884	86.02 ± 19.12	0.132	77.62 ± 29.22	0.651
	NMCPK	64.25 ± 30.41		74 ± 31.59		70.3 ± 32.95	
Appearance average	MCPK	68.60 ± 21.71	0.847	78.48 ± 19.64	0.129	81.81 ± 19.28	0.500
	NMCPK	66.16 ± 20.63		69.01 ± 20.77		74.8 ± 19.37	
Residual limb average	MCPK	74.71 ± 14.12	0.613	76.46 ± 22.57	0.053	72.8 ± 21.27	0.564
	NMCPK	68.58 ± 21.55		62.63 ± 23.46		79.47 ± 22.08	
Frustration average	MCPK	60 ± 44.16	0.216	90.05 ± 15.7	0.049	87.69 ± 26.31	0.217
	NMCPK	81.50 ± 19.59		76.43 ± 27.34		67 ± 40.87	
Perceived response average	MCPK	85.42 ± 14.62	0.456	93.41 ± 10.89	0.116	90.65 ± 15.92	0.612
	NMCPK	90.92 ± 11.07		86.36 ± 17.21		85.75 ± 23.05	
Social burden average	MCPK	97.08 ± 5.83	0.232	86.71 ± 20.5	0.623	87.95 ± 15.44	0.468
	NMCPK	84.44 ± 19.17		83.62 ± 20.84		76.4 ± 31.33	
Ambulation average	MCPK	58.54 ± 31.28	0.289	80.28 ± 20.46	0.001	73.34 ± 20.52	0.140
	NMCPK	72.2 ± 15.87		54.09 ± 25.83		56.03 ± 23.12	
Well-being average	MCPK	88 ± 8.12	0.776	87.36 ± 18.58	0.263	82.27 ± 19.56	0.938
	NMCPK	85.85 ± 13.64		79.48 ± 26.37		81.3 ± 31.55	
Total average score	MCPK	74.91 ± 16.9	0.794	80.34 ± 15.26	0.012	81.1 ± 14.18	0.447
	NMCPK	76.46 ± 5.72		72.19 ± 15.23		73.85 ± 25.39	

**Table 4:** PEQ for genders based on prosthetic knee joint type PEQ: Prosthetic Evaluation Questionnaire, MCPK: microprocessor-controlled prosthetic knee, NMCPK: non-microprocessor-controlled prosthetic knee

PEQ	Prosthetic knee	Male		Female	
		Mean ± Std. Deviation	P value	Mean ± Std. Deviation	P value
Utility average	MCPK	78.62 ± 17.12	0.048	76.66 ± 4.37	0.230
	NMCPK	68.58 ± 23.06		66.17 ± 18.39	
Sounds average	MCPK	79.54 ± 27.31	0.126	89 ± 12.5	0.781
	NMCPK	68.36 ± 31.21		84.75 ± 27.34	
Appearance average	MCPK	79.8 ± 19.76	0.047	68.18 ± 15.82	0.791
	NMCPK	69.83 ± 20.19		64.72 ± 21.45	
Residual limb average	MCPK	76.31 ± 20.93	0.051	64.13 ± 21.09	0.515
	NMCPK	65.32 ± 24.01		72.21 ± 16.54	
Frustration average	MCPK	88.01 ± 21.97	0.072	62.83 ± 45.95	0.662
	NMCPK	76.95 ± 27.06		74.25 ± 30.14	
Perceived response average	MCPK	92.54 ± 12.85	0.359	83.81 ± 13.8	0.621
	NMCPK	89.5 ± 13.85		76.67 ± 25.02	
Social burden average	MCPK	89.28 ± 17.1	0.234	79.25 ± 23.99	0.976
	NMCPK	83.65 ± 20.73		78.72 ± 27.02	
Ambulation average	MCPK	76.57 ± 22.85	0.007	67.47 ± 13.1	0.258
	NMCPK	60.1 ± 24.98		53.83 ± 19.44	
Well-being average	MCPK	86.62 ± 17.96	0.521	77.75 ± 18.21	0.680
	NMCPK	83.33 ± 23.29		71.08 ± 27.05	
Total average score	MCPK	83.03 ± 15.37	0.015	74.56 ± 7.81	0.763
	NMCPK	73.93 ± 14.2		71.38 ± 19.06	

**Table 5:** PEQ for causes of amputation based on prosthetic knee joint type PEQ: Prosthetic Evaluation Questionnaire, MCPK: microprocessor-controlled prosthetic knee, NMCPK: non-microprocessor-controlled prosthetic knee

PEQ	Prosthetic knee	Traumatic cause		Disease cause		Congenital cause	
		Mean ± SD	p-value	Mean ± SD	p-value	Mean ± SD	p-value
Utility average	MCPK	76.13 ± 17.03	0.169	82.41 ± 12.62	0.074	100 ± 0	0.211
	NMCPK	67.7 ± 25.1		69.17 ± 18.56		68.3 ± 19.47	
Sounds average	MCPK	77.65 ± 28.84	0.441	88.9 ± 17.72	0.550	75 ± 0	0.432
	NMCPK	71 ± 30.9		83.36 ± 23.21		43.4 ± 33.02	
Appearance average	MCPK	75.37 ± 20.39	0.208	85.59 ± 15.88	0.171	95 ± 0	0.143
	NMCPK	67.63 ± 22.03		75.25 ± 17.29		61.45 ± 16.86	
Residual limb average	MCPK	72.24 ± 22.66	0.322	80.06 ± 14.45	0.546	100 ± 0	0.081
	NMCPK	65.7 ± 22.78		74.93 ± 22.44		50.77 ± 19.39	
Frustration average	MCPK	86.35 ± 23.66	0.162	83.6 ± 29.17	0.826	100 ± 0	0.435
	NMCPK	75.66 ± 28.51		81 ± 24.37		70.5 ± 31.04	
Perceived response average	MCPK	91.44 ± 13.82	0.414	91.28 ± 11.78	0.640	100 ± 0	0.543
	NMCPK	88.27 ± 12.77		87.73 ± 20.74		83.4 ± 22.84	
Social burden average	MCPK	86.46 ± 19.62	0.665	91.83 ± 12.36	0.411	100 ± 0	0.459
	NMCPK	83.97 ± 19.69		85.33 ± 21.4		72.67 ± 30.49	
Ambulation average	MCPK	74.19 ± 24.5	0.158	77.01 ± 14.06	0.002	100 ± 0	0.185
	NMCPK	64.06 ± 24.79		48.25 ± 21.69		61.23 ± 22.12	
Well-being average	MCPK	85.35 ± 19.68	0.744	85.15 ± 13.54	0.570	100 ± 0	0.484
	NMCPK	83.32 ± 23.61		80.09 ± 24.47		75.8 ± 28.66	
Total average score	MCPK	80.51 ± 16.38	0.149	85.09 ± 9.93	0.152	96.67 ± 0	0.159
	NMCPK	74.11 ± 13.59		76.13 ± 16.43		65.28 ± 16.59	

## Discussion

The self-reporting questionnaire for the scales reflected the participants' understanding of the value of MCPK for ambulation in different services, general fitting, utility, and appearance when it includes microprocessor technology, although it was not reflected in frustration, social burden, perceived response, and well-being. The PEQ for ambulation includes eight questions about walking with a prosthesis, walking in a small space, walking upstairs and downstairs, walking up and down hills, walking on a sidewalk, and walking on slick surfaces. While many articles compared each question individually, all were reported as a total score under ambulation [15]. Other articles reviewed mobility and gait variation together [16].

The study results confirm that users can detect an increase in ambulation when using MCPK versus NMCPK, correlating with previous study findings of increased self-reported ambulation and satisfaction [17-21]. Simultaneously, other studies [2,8] reported comparable step counts based on duration. The improvement in utility and appearance in MCPK users was significant, and the same findings were reported in the literature [7]. The number of female participants was not enough to report any significance, while the male results of the significance of MCPK are the same with all participants. In comparison to early adulthood (18-24 years) and late adulthood (41-60 years), MCPK users in middle adulthood (25-40 years) had substantial differences in utility, frustration, ambulation, and total PEQ score. These findings are consistent with other research that shows that the value of dissatisfaction in this age group is higher than in early and late adulthood. This age group has recognized the benefits of MCPK in lowering frustrations [22] (Table 3).

Chronic diseases such as diabetes and necrosis as a cause of amputation differed significantly in ambulation for MCPK users. There were no significant findings in traumatic and congenital causes of amputation. In MCPK, amputation due to trauma or congenital reasons had a lower value of ambulation than amputation due to disease. Participants with disease-related amputations obtained self-reported ambulation in several services and a walking level not possible with NMCPK. Compared to patients with amputations caused by diseases and congenital reasons, patients with amputations caused by trauma typically seek a higher level of ambulation. The well-being scale showed insignificant improvement in all categories (age, gender, and cause of amputation), which agrees with the other findings of the same comparison [7]. Higher prosthetic satisfaction [23,24] improved quality of life and body image [25,26], and greater well-being was noted with MCPK when compared to NMCPK [24].

Comparing two types of MCPK, C-Leg and Genium, using PEQ, the responses for social burden, utility, and well-being reveal a significant advantage for Genium. Mobility, ADL performance, and quality of life have all improved significantly with Genium. These results back up the findings of the Genium knee joint when compared to the MCPK C-Leg [9]. The use of various satisfaction questionnaires made comparing the results difficult. The original PEQ scales were used, as well as its modified versions, such as PEQ-MS 13/11, PEQ-MS 12/5, and PEQ-MS 13/7 [27,28]. The study suggests MCPK prostheses improve gait, daily activities, and overall experience in transfemoral amputees; however, the authors would like to acknowledge some limitations in this section. Firstly, the modified PEQ scale employed in this study made it a little difficult to compare our results with previously published literature as this was a novel modification of the PEQ scale employed in the earlier studies. Secondly, as the study is done in a homogeneous population, the results obtained should be used with caution, and similar studies need to be done in other populations as well. Lastly, reporting PEQ individual questions rather than the scale may also impede proper analysis of the findings. More research is needed to focus on the outcome of MCPK in order to find a link between demographic information and prosthetic knee joint selection criteria.

There is no evidence within the body of literature that NMCPK improves clinical outcomes compared to MCPK. There is limited evidence that MCPK adds value when compared to NMCPK. Various levels of evidence suggest that different types of MCPK result in different outcomes for unilateral transfemoral amputees compared to NMCPK. More research is required to emphasize the outcome of MCPK to discover a link between demographic information and prosthetic knee joint selection criteria.

## Conclusions

Improvement in transfemoral amputees using MCPK prostheses in terms of expression in quality of life increases the expectations of prosthetic rehabilitation. These prostheses were also associated with improved performance in gait and daily activities throughout our study period. MCPK improves the transfemoral amputee's overall experience among different gender, age categories, and etiologies. Considering the limitations of this study, further studies with larger sample sizes that evaluate the effects of a variety of previously used NMCPK should be conducted to check the efficacies of different MCPK types further.
